# Performance evaluation of machine learning-based infectious screening flags on the HORIBA Medical Yumizen H550 Haematology Analyzer for vivax malaria and dengue fever

**DOI:** 10.1186/s12936-020-03502-3

**Published:** 2020-11-23

**Authors:** Parag Dharap, Sebastien Raimbault

**Affiliations:** 1 Dharap’s Diagnostic Center, Mumbai, India; 2 HORIBA Medical, Montpellier, France

## Abstract

**Background:**

Automated detection of malaria and dengue infection has been actively researched for more than two decades. Although many improvements have been achieved, these solutions remain too expensive for most laboratories and clinics in developing countries. The low range HORIBA Medical Haematology Analyzer, Yumizen H550, now provides dedicated flags ‘vivax malaria’ and ‘dengue fever’ in routine blood testing, developed through machine learning methods, to be used as a screening tool for malaria and dengue fever in endemic areas. This study sought to evaluate the effectiveness of these flags under real clinical conditions.

**Methods:**

A total of 1420 samples were tested using the Yumizen H550 Haematology Analyzer, including 1339 samples from febrile patients among whom 202 were infected with malaria parasites (*Plasmodium vivax* only: 182, *Plasmodium falciparum* only: 18, both: 2), 210 were from febrile dengue infected patients, 3 were from afebrile dengue infected patients and 78 were samples from healthy controls, in an outpatient laboratory clinic in Mumbai, India. Microscopic examination was carried out as the confirmatory reference method for detection of malarial parasite, species identification and assessing parasitaemia based on different stages of parasite life cycle. Rapid diagnostic malarial antigen tests were used for additional confirmation. For dengue infection, NS1 antigen detection by ELISA was used as a diagnostic marker.

**Results:**

For the automated vivax malaria flag, the original manufacturer’s cut off yielded a sensitivity and specificity of 65.2% and 98.9% respectively with the ROC AUC of 0.9. After optimization of cut-off value, flag performance improved to 72% for sensitivity and 97.9% specificity. Additionally it demonstrated a positive correlation with increasing levels of parasitaemia. For the automated dengue fever flag it yielded a ROC AUC of 0.82 with 79.3% sensitivity and 71.5% specificity.

**Conclusions:**

The results demonstrate a possibility of the effective use of automated infectious flags for screening vivax malaria and dengue infection in a clinical setting.

## Background

### Malaria

Malarial diagnosis is routinely performed with the microscopic examination of thick or thin stained blood smears, a task both time consuming and requiring an experienced microscopist. Immunochromatographic pan-*Plasmodium* pLDH antigen detection-based rapid diagnostic tests (RDT) with or without sub-species specific detection system are also used, but are expensive, vulnerable to temperature and humidity as well as demonstrating variable sensitivity and specificity. Other modalities, such as specific PCR [1], flow cytometry-based parasite DNA or RNA detection [2, 3] have been developed, but are time consuming, non-affordable, non-automated and therefore are considered impractical for routine application in endemic countries.

### Automated malaria detection

Automated digital microscopy and cell classification with CellaVision (Lund, Sweden) DM96 Advanced RBC Application in the fully automated mode, showed high specificity; however, this has been reported to give a sensitivity of only 23% [4]. The same image classification software is used in the Sysmex (Kobe, Japan) DI-60, where sensitivity has been found to be similar to microscopy, but only after manual human reclassification [5]. False negative results are still seen, even for 6.3% parasitaemia, possibly because these systems are very sensitive to smear quality and staining [6]. Furthermore, these systems provide a general parasite class without *Plasmodium* species identification, again unable to circumvent the need for skilled morphologists.

Haematology Analyzers reporting CBC/DIFF have also been used for malarial screening. Parameters frequently used for screening, such as platelet and WBC counts, may also be affected in diseases other than malaria and hence provide poor sensitivity and specificity. These reported malarial algorithms still involve some mathematical calculation and require interpretative skills to assess laboratory results.

During intra-erythrocytic stages, a malarial parasite in the RBC digests haemoglobin and releases free haem, which is toxic to cells, further converting it into a bi-refringent insoluble crystalline form called haemozoin [7]. Abbott (Santa Clara, USA) designed the Cell-Dyn series with a depolarized diffracted light (DLL) detector, therefore malaria cases could be flagged by cells identified as large mononuclear cells with a high depolarized signal due to haemozoin [8, 10]. This detection method lacks sensitivity for early infection, as significant haemozoin production occurs only with late stages or mature Plasmodium parasite forms. Regarding specificity, false positives have been reported in cases of other parasites, such as filaria [9], or children treated with sulfonamide, as its derivatives form yellow granules that give a similar depolarized signal [10]. Varying performance has been reported among studies [10] and among instruments, depending on laser wavelengths. Detection of haemozoin has also been proposed using a magneto-optical device [11].

In infected red blood cells (iRBCs), the cytoskeleton is remodelled by the *Plasmodium* [12–14], accompanied by changes in iRBC membrane properties. Although not yet clearly understood, these changes can result in an increased resistance to lysis for iRBCs, especially those harbouring mature forms of the parasite (late amoeboid, schizonts, gametocytes). These incompletely lysed iRBC may then produce a spurious signal in WBC channels, as a peak on the left of WBC volume histogram or as a separate cluster [15]. Since this process is lysis-dependent, balance alarms are often triggered by differences between WBC counting channels using different lysis methods.

Detecting iRBCs has been used for malaria detection with Sysmex XE [16–18], XN series [19] and Mindray (Shenzhen, China) BC-6800 [20]. This technique lacks sensitivity for low levels of parasitaemia and in early infection, as only mature forms are likely to have remodeled the cytoskeleton sufficiently to increase lysis resistance. Furthermore, while *Plasmodium vivax* remodelling results in a more flexible (deformable) membrane of the iRBC in order to avoid splenic entrapment, the presence of *Plasmodium falciparum* conversely affects iRBC by increasing membrane rigidity [21]. The *P. falciparum* survival strategy is to increase iRBC membrane adherence and sequestration in venules and capillaries [22]. This may explain the scarcity of finding mature forms of *P. falciparum* in venous blood, thus resulting in less sensitivity for those Analyzers. Recently, Sysmex has released the high range XN-30 Analyzer, embedding a 405 nm violet laser with scattering and fluorescence measurements, that has a dedicated module (for additional cost) for *Plasmodium* detection and counting with a partial lysis reagent allowing parasites to remain inside iRBCs and nucleic acid staining for labelling parasites DNA, which claims an improved detection limit of 20 parasites/µL [23]. This device has been evaluated for malaria detection, reporting a ROC with an AUC = 0.98 and achieving 98.7% sensitivity and 96.5% specificity when compared with microscopy reference [24, 25].

Another technique was developed using various discriminant factors based on statistical variables of cell population data (CPD) for high range Beckman-Coulter instruments using VCS technology to identify malarial infection. Standard deviations of the volume and conductivity of lymphocytes has been used along with PLT count [26], or the standard deviation of the volume of lymphocytes and monocytes [27]. Using CPD on the Sysmex XN, Buoro et al*.* found 93% sensitivity, but employing both techniques, an AUC of 0.96 was achieved with sensitivity 100% and specificity 91%, albeit with a limited study of only 14 positive cases [19].

All of these techniques developed to date have been on high range instruments using laser-technology that are generally not in routine use in endemic regions of developing countries where field laboratories typically have low or middle range instruments, thus leaving a persistent need for affordable screening tools. In a previous work [28], the complete data set was generated using two Haematology Analyzers (ABX Pentra XL80, 5-Diff, HORIBA Medical, Montpellier, France and Microsemi CRP, 3-Diff + CRP, HORIBA Medical, Kyoto, Japan) and explored automated SVM classifiers for malaria with data-mining techniques customized for each instrument. The flags generated by these classifiers were evaluated in a prospective study and showed similar performance to claims for malarial flags or algorithms on high range instruments. For the Micros-EMI-CRP, the inclusion of CRP in the flagging algorithm provided valuable information for malaria screening, increasing sensitivity beyond only the CBC data. Unfortunately, the addition of a CRP value to a malarial flagging algorithm does not allow for a cost-effective malarial screening tool.

### Dengue fever

Dengue virus is an arbovirus transmitted by *Aedes* mosquitoes and exists as four serotypes, DENV-1 to 4 [29]. Dengue infection can range from simple fever to more severe cases with lethal bleeding tendencies due to thrombocytopenia and in the most serious cases with plasma leakage, referred as severe dengue fever. As opposed to malaria where haemozoin or lysis-resistant iRBCs can be observed, there is no obvious specific cellular signal indicative of dengue virus infection in standard Haematology Analyzers. Only the non-specific host immunologic responses common to many viral infections can be observed.

Some effects on haematology parameters, such as lymphocytes, neutrophils and monocytes, have been reported in dengue infections [30, 31], which correlate with the number of days of illness and thus are not so effective for screening in early stages of the disease [32].

Some previous studies have defined certain discriminant factors for dengue screening, such as the one developed using CPD on Beckman-Coulter VCS instruments. A review of literature in relation to discriminating dengue fever from other febrile illness can be found in the references [30]. Algorithms distinguishing malaria and dengue fever from other febrile conditions have been designed using the PLT count [26] or via the percentage of lymphocytes and standard deviation of the conductivity of lymphocytes on the LH750, reporting an AUC of 0.893, also evaluated with an AUC of 0.931 in reference [15]. Soto et al. reported AUC of 0.76 for dengue factor built from the percentage of monocytes and the standard deviation in the volume of monocytes [33]. The lymph-index, defined as lymphocyte mean volume x SD of lymphocyte volume/lymphocyte mean conductivity, has been designed as a factor for viral infection for the LH750, reporting a potential sensitivity of 71% and specificity of 78% for dengue fever detection [34, 35]. Dengue factor and lymph-index factor have also been evaluated with 2 new factors: Monocyte Factor from Mono% and SD of Monocyte volume, and a new dengue factor mixing monocyte factor, lymph-index and platelet (PLT) count [36], but none of these achieved a ROC AUC greater than 0.70. A decision tree designed with the same variables with the LH780 has been reported to achieve a sensitivity of 94% and specificity of 77% for suspected samples [37]. Two decision trees (one of these only using Haematology Analyzer parameters) for classifying severity of the disease have been proposed [38].

## Methods

The purpose of this study was to evaluate the performance of malaria and dengue suspicion flags incorporated into a new generation of low to middle range Haematology Analyzer, the Yumizen H550 by HORIBA Medical, Montpellier, France. Additionally, the achievable performance using further optimized cut-off values was retrospectively evaluated.

### Machine learning predictors

A definite need for automated screening technology has been identified in areas endemic for malaria and dengue. These are largely located in developing countries which, being extremely cost-sensitive, frequently employ low or mid-range analyzers possessing non-laser technology with limited spectral detection capabilities and reagent complexity. Although these low-cost technologies generate limited information for detection, data acquired in such a Haematology Analyser in a single sample run can still range from a hundred to tens of thousands of variables. This work hypothesized that this data may yet have the potential for building an automated classifier algorithm with help of machine learning methods. In this study, two approaches were used. The first was to build the suspicion flags from the raw acquisition data alone in order to avoid revalidation after a change in a single algorithm. While this approach lacks information, such as CPD requiring prior cell classification, it makes the flags generated independent from other computations.

With the second approach, in order to have greater reproducibility between instruments and reagent batches, the measurements were binned, for example a 128 channels histogram being converted into 16 channels, each derived by the binning of 8 channels.

A set of 243 samples were tested in India in 2017 using reference methods for the target diagnosis (malaria: RDT + thin smear, dengue: ELISA) and processed in duplicate on the Yumizen H550. Various types of classifiers were evaluated with cross-validation techniques, but random forests (using ranger-ml implementation [39]) were found to perform best for these flags, using 1000 trees per forest. Subsequently, a subset of *n* = 393 variables were selected with cross-validation, discarding the variables having no relevant information for malaria nor dengue. Instead of classic random forests, classification models where each node has only √*n*≈20 variables were used, regression models using *n*/3 = 131 variables for splitting a node were trained to predict probability scores (range [0–1]) of infection with *P. vivax* (probPV) or dengue (probDengue), in order to ease cut-off tuning (default cut-offs being 0.5). Finally, the models were retrained with the whole set of data before evaluation of the prototype software commenced.

### Samples

EDTA anti-coagulated blood samples were collected from patients in Dharap’s Diagnostic Center, Mumbai, India between July and October in 2018. The specimens and data were collected in a coded fashion in accordance with local ethical or IRB guidelines and to national and international standards for conducting clinical studies including 21CFR Parts 50 and 56 and International Conference on Harmonization (ICH) E6 –Good Clinical Practice Consolidated Guideline.

The patients’ febrile presentation was included, resulting in a febrile group of 1339 samples, an afebrile dengue positive group of three samples and a control non-febrile group of 78 normal samples, totaling 1420 samples (M/F ratio: 56.2%/43.8%, age range: 1–96 years, grouped as [≤ 12: Child, ≥ 60: Elderly, Adult otherwise]).

Among the 1339 febrile patients samples (age: 1–96, M/F: 57%/43%), 202 were malaria parasite-infected (age: 5–78, M/F: 67.3% / 32.7%, *P. vivax* only: 182, *P. falciparum* only: 18, both: 2), 210 were dengue infected (age: 2–90 years, M/F: 61%/39%) and 3 samples were concomitantly infected by dengue and vivax malaria.

Thin and thick blood films were prepared for staining and parasite investigation. All the blood smears were examined using a light microscope (Olympus CH 30i) by an experienced microscopist blinded to the Analyzer flag results.

Microscopic examination of the stained thin smear was conducted as the reference method for identification of *Plasmodium* species, parasite stages and assessing parasitaemia which was estimated by counting 2000 RBCs. Rapid malaria antigen detection test kits (SD Biosensor Healthcare Pvt. Ltd., Gurugram, India) using monoclonal anti-*P. falciparum* HRP-II (0.75 + / − 0.15 µg) with monoclonal anti-*P. vivax* pLDH (0.75 + / − 0.15 ug) respectively, were used additionally to detect and confirm the presence of *P. falciparum* & *P. vivax* related antigens.

For screening and diagnosis of dengue fever, RecombiLISA NS1 Antigen test was carried out, utilizing pairs of specific polyclonal & monoclonal anti-dengue antibodies of all four serotypes (DEN1, 2, 3, 4) with analytical sensitivity of 0.3 ng/ml for type 2 NS1 antigen (CTK Biotech, Inc., Poway, Ca USA).

All samples were processed in duplicate (2840 total runs) on the Yumizen H550 instrument, using the manufacturer recommended reagents, calibrator and controls. Analyzer performance was monitored daily using three levels of quality control material. Calibration was completed at the start of the study and confirmed at the conclusion of the study.

**Fig. 1:**
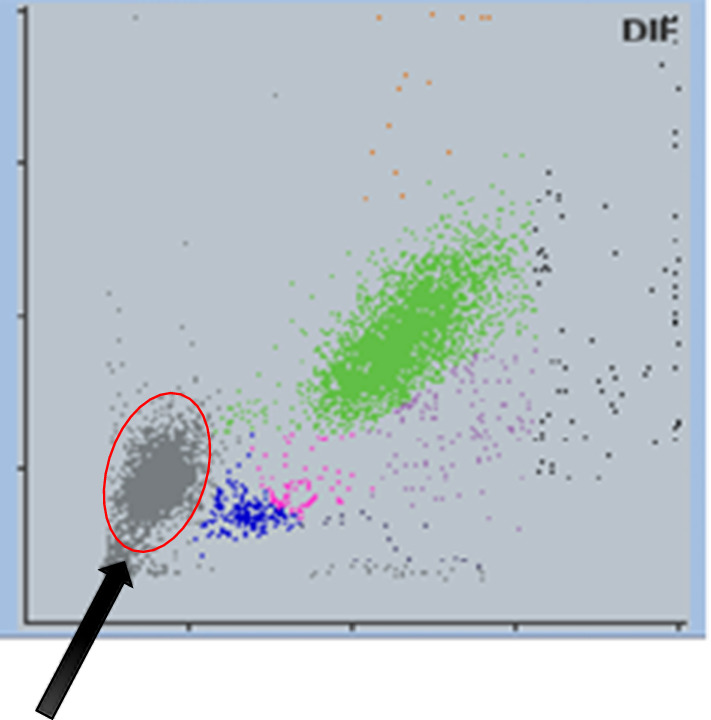
Cluster of iRBCs containing malarial forms in WBC matrix

Yumizen H550 is a low range 5-diff automated Analyzer embedding a mini flowcell for WBC counting and differential, in addition to RBC/PLT channels for counting and volume measurement by impedance and a HGB channel. The WBC channel provides a volume/scattering matrix where a cluster of events can be seen, mainly with mature forms of *P. vivax* (Fig. 1).

### Data analysis

Data analysis was performed using Microsoft Office Excel. Statistical analysis was performed using Analyse-It 4.65.2 (Analyse-it Software Ltd, UK) for Excel. ROC analysis with Wilcoxon-Mann–Whitney AUC estimator and Delong et al. 95% CI, sensitivity and specificity with Wilson 95% CI and predictive values with Mercado-Wald 95% CI for the detection of *P. vivax* or dengue fever were calculated.

## Results

**Fig. 2:**
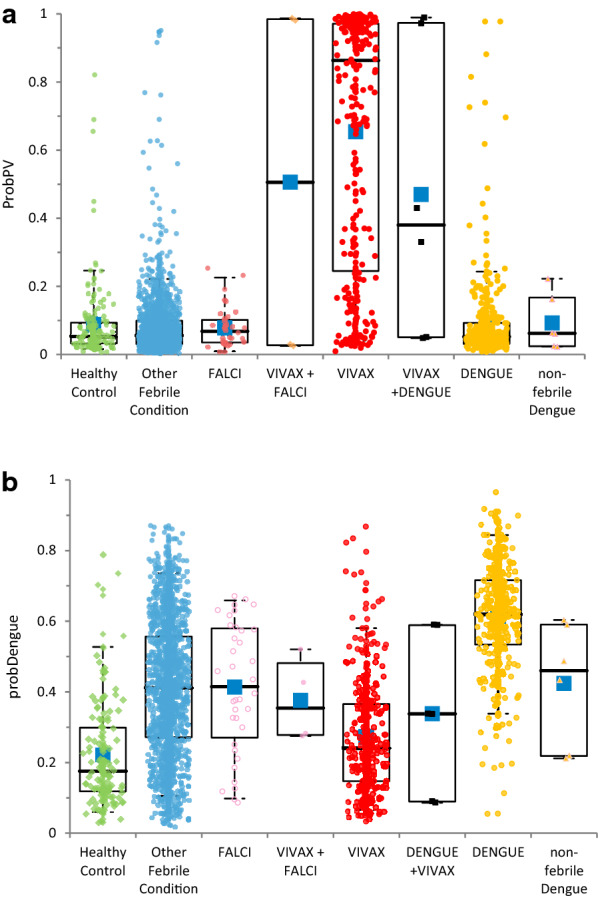
Distribution of scores for all groups. **a** ProbPV. **b** ProbDengue. Box plot legend: plain box: q1–q3 quartiles, dash-line: 5–95% quantiles, bold line: median, blue square: mean

Figure 2 shows the distribution for each group of computed scores probPV (vivax) and probDengue. Mean absolute difference between duplicates on [0–1] scale were 0.0277 (95% < 0.10) and 0.0445 (95% < 0.13), respectively. The ROCs of first and second replicates showed no significant difference for each score.

Table 1 shows the performance of the flags for all samples, for febrile patients, for malaria and/or dengue positive patients, and finally for malaria positive patients in order to evaluate *P. vivax* identification ability of probPV. The possible performance with a retrospectively optimized vivax malaria flag cut-off of 0.31 is also presented.

**Table 1:** Diagnostic performance of vivax malaria and dengue fever flags

			Overall	Febrile	Malaria or dengue infected	Malaria infected
Nsamples			1422	1341	412	202
Vivax malaria	ROC AUC		0.902 [0.880–0.924]	0.902 [0.880–0.924]	0.900 [0.877—0.923]	0.901 [0.868—0.933]
	cut-off 0.5	Sensitivity	65.2% [60.2–69.9%]	65.2% [60.2–69.9%]	65.2% [60.2–69.9%]	65.2% [60.2–69.9%]
		Specificity	98.9% [98.5–99.3%]	99% [98.5–99.3%]	98.2% [96.6–99.1%]	100% [90.4 – 100%]
		PPV	90.2% [86.2–93.2%]	91.3% [87.3–94.0%]	96.8% [93.8–98.4%]	100% [ -]
		NPV	95% [94.3–95.6%]	94.7% [94.0–95.4%]	77.8% [75.3–80.1%]	22% [19.6–24.4%]
		Younden	0.642	0.642	0.635	0.652
	cut-off 0.31	Sensitivity	72.0% [67.2–76.4%]	72.0% [67.2–76.4%]	72.0% [67.2–76.4%]	72.0% [67.2–76.4%]
		Specificity	97.9% [97.3–98.4%]	98% [97.4–98.5%]	96.9% [94.9–98.2%]	100% [90.4 – 100%]
		PPV	83.9% [79.7–87.3%]	85.2% [81.1–88.5%]	95.0% [91.8–97.0%]	100% [ -]
		NPV	95.9% [95.2–96.5%]	95.6% [94.9–96.3%]	81.1% [78.4–83.5%]	25.9% [22.9–29.2%]
		Younden	0.699	0.700	0.689	0.720
Dengue	ROC AUC		0.814 [0.793–0.834]	0.808 [0.787–0.829]	0.904 [0.883–0.926]	-
	cut-off 0.5	Sensitivity	79.3% [75.2–82.9%]	80.0% [75.9–83.5%]	79.3% [75.2–82.9%]	-
		Specificity	71.6% [69.8–73.3%]	70.1% [68.1–71.9%]	86.4% [82.7–89.5%]	-
		PPV	33.0% [31.3–34.8%]	33.2% [31.5–35.0%]	86.2% [82.9–89.0%]	-
		NPV	95.2% [94.2–96.0%]	95.0% [93.9–95.8%]	79.6% [76.4–82.5%]	-
		Younden	0.509	0.501	0.658	-

### Vivax malaria flag

Some difference in performance between genders was found. (M/F samples: 799/621, vivax malaria positive ratio 15.8%/10.0%). ROC AUC are 0.904 for males and 0.895 for females. The following findings support the hypothesis that this might be linked to differences in parasitemia (M/F: mean = 4.37/3.66, median = 2.5/2.0, per 1000 RBCs).

Sensitivity was better for males than females (M/F prospective cut-off 0.5: 68.9%/58.1%, retrospectively optimized cut-off 0.31: 75.0%/66.1%). ProbPV scores means and medians were higher for vivax positive males (M/F: N = 240/124, mean = 0.68/0.59, median = 0.90/0.76).

**Fig. 3:**
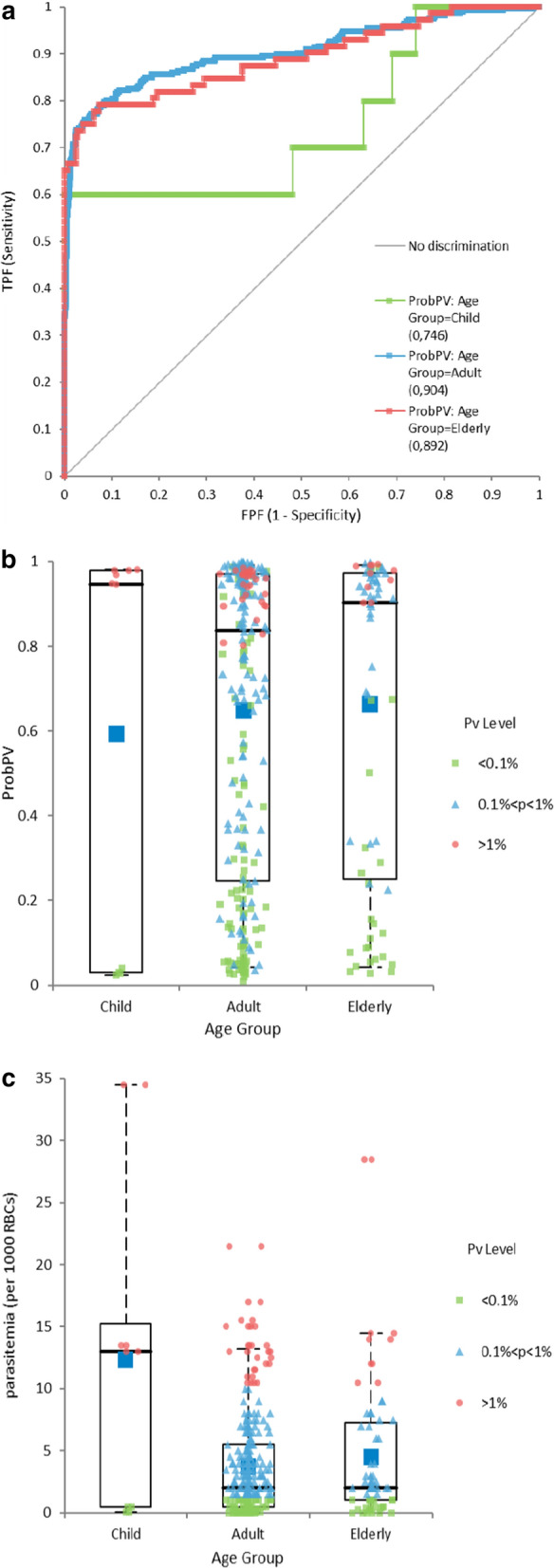
Vivax detection by age group. **a** ProbPV ROCs by age group. **b** Vivax positive ProbPV values by age group. **c** Vivax positive parasitaemia by age group

Some differences were also seen depending on age (Fig. 3). The age groups had different sizes (Child: 165, Adult: 1075, Elderly: 180) and vivax positive ratios (Child: 3.03%, Adult: 13.3%, Elderly: 20%).

Performance was lower for children than for adults, but this is likely a function of the limited number of samples, with only 5 vivax positive children, among which 2 with high parasitemia were detected and 3 with low parasitemia were not detected. ROC for group Elderly showed AUC slightly lower than for adults, although parasitemia was marginally higher for this group.

**Fig. 4:**
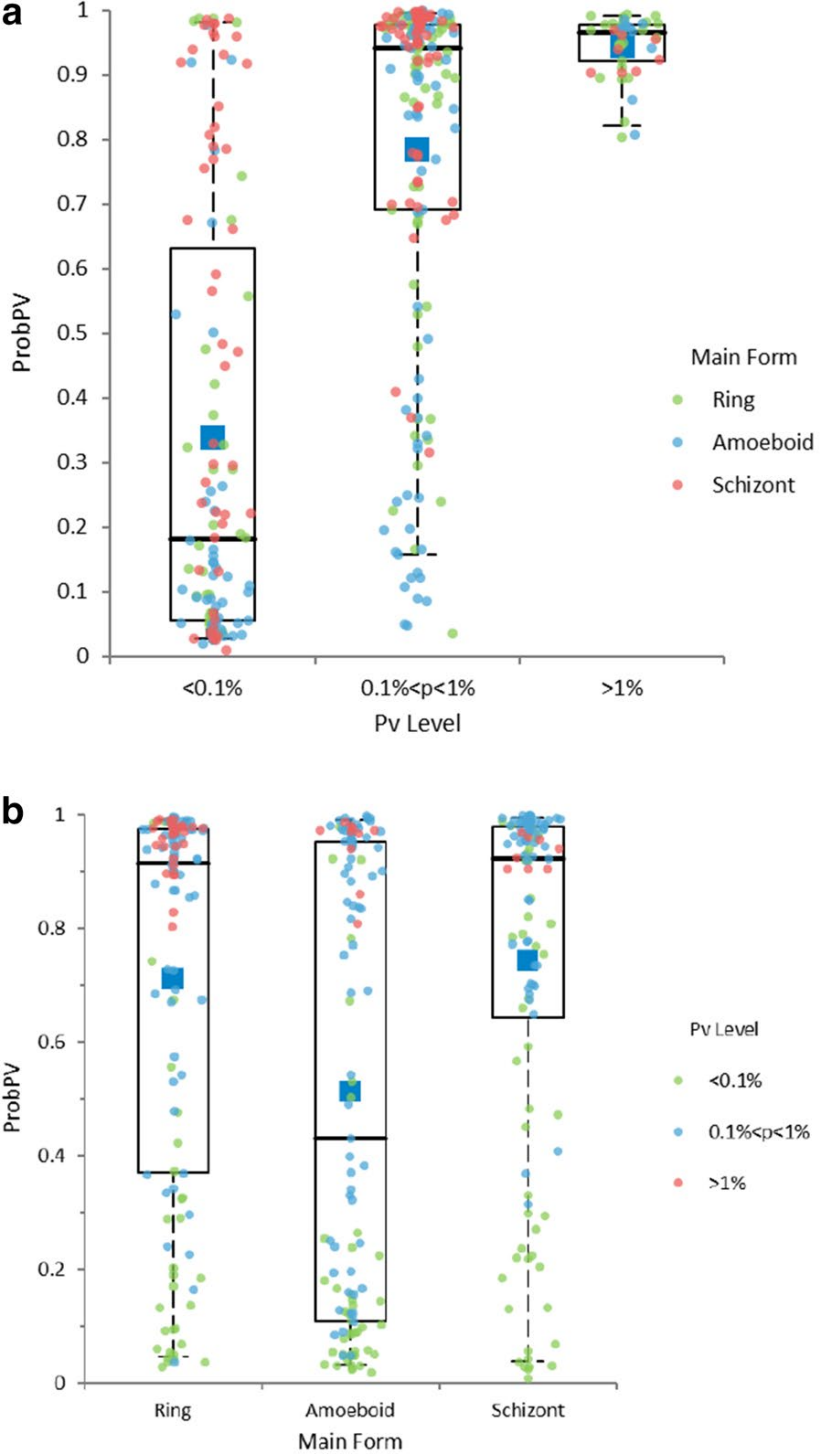
Vivax detection by parasitaemia and predominant form. **a** Vivax positive ProbPV values by parasitaemia groups. **b** Vivax positive ProbPV values by predominant form

Performance of Pv detection was clearly linked to parasitaemia level. *Plasmodium vivax* positive samples were classed into 3 parasitaemia groups. For the low parasitaemia group (p < 0.1%RBC, N = 64), sensitivity with default cut-off 0.5 was only 28.9% and 35.9% with optimized cut-off 0.31. For the medium parasitaemia group (0.1%RBC < p < 1%RBC, N = 97), sensitivity with default cut-off 0.5 was 80.9% with default cut-off and 89.2% with an optimized cut-off. For the high parasitaemia group (p > 1%RBC, N = 23), sensitivity was 100% with both cut-offs. Figure 4a shows the values of *probPV* for vivax positive samples among these groups.

The detection rate was also dependent on the predominant forms of the *Plasmodium*. For rings, sensitivity was 69.8% with cut-off 0.5, and 77.5% with optimized cut-off 0.31. For amoeboid trophozoites, sensitivity was only 47.2% with cut-off 0.5, and 53.6% with optimized cut-off 0.31. For schizonts, sensitivity was 74.6% with cut-off 0.5, and 80.3% with optimized cut-off 0.31. Figure 4b shows the values of *probPV* for vivax positive samples among these groups. The detection rate was much better for schizonts, as these mature forms typically produce more lyse resistance and thus give a stronger signal in the iRBC cluster of the WBC matrix (Fig. 1). However, it is unclear why detection rate was better when the predominant form was ring rather than amoeboid.

### Dengue fever flag

**Fig. 5:**
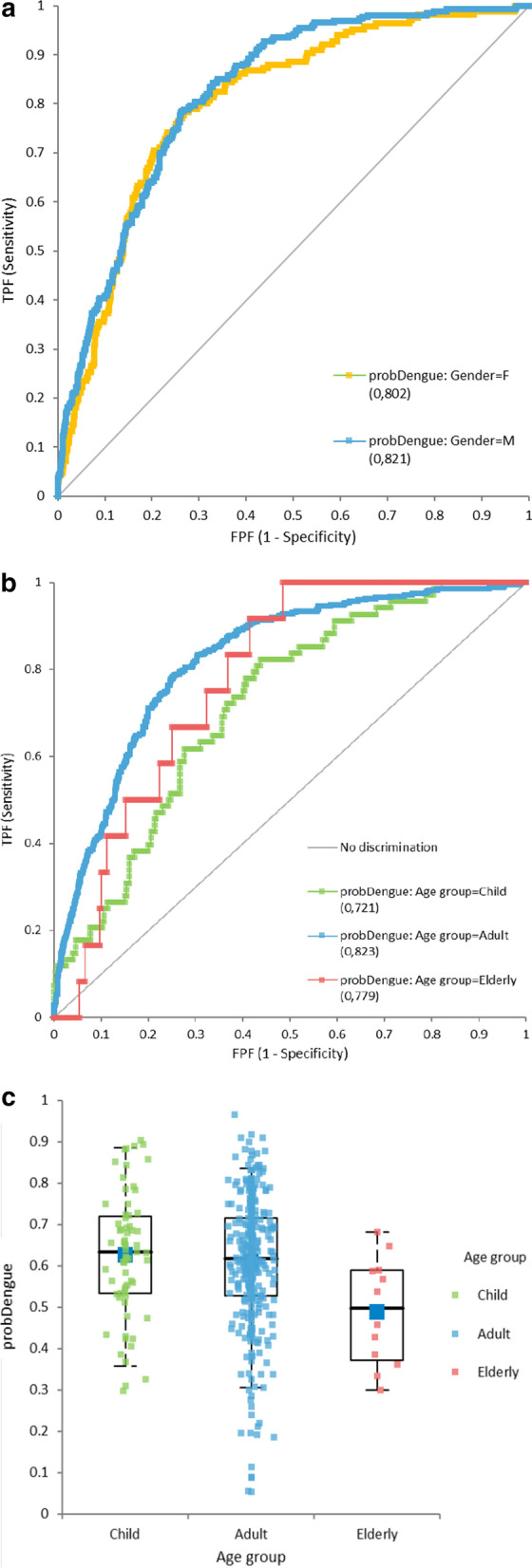
Dengue detection by gender and age group. **a** ProbDengue ROCs by gender. **b** ProbDengue ROCs by age group. **c** Dengue positive ProbDengue values by age group

Some difference in performance associated with gender was also found among the dengue fever patients (F/M samples: 621/799, dengue fever positive ratio 13.4%/16.3%) (Fig. 5a). ROC AUC were 0.802 for females and 0.821 for males (Fig. 5a). Sensitivity was slightly better for males (79.6%) than females (78.9%) with same specificity (71.6%).

Some differences were also seen depending on age (Fig. 5b). The age groups had different sizes (Child: 165, Adult: 1075, Elderly: 180) and dengue fever positive ratios (Child: 18.5%, Adult: 16.4%, Elderly: 3.3%). For the paediatric group, the lowest specificity was observed. For elderly group, there was a limited number of positive samples (6 so only 12 runs), but probDengue values were lower (Fig. 5c).

## Discussion

**Fig. 6:**
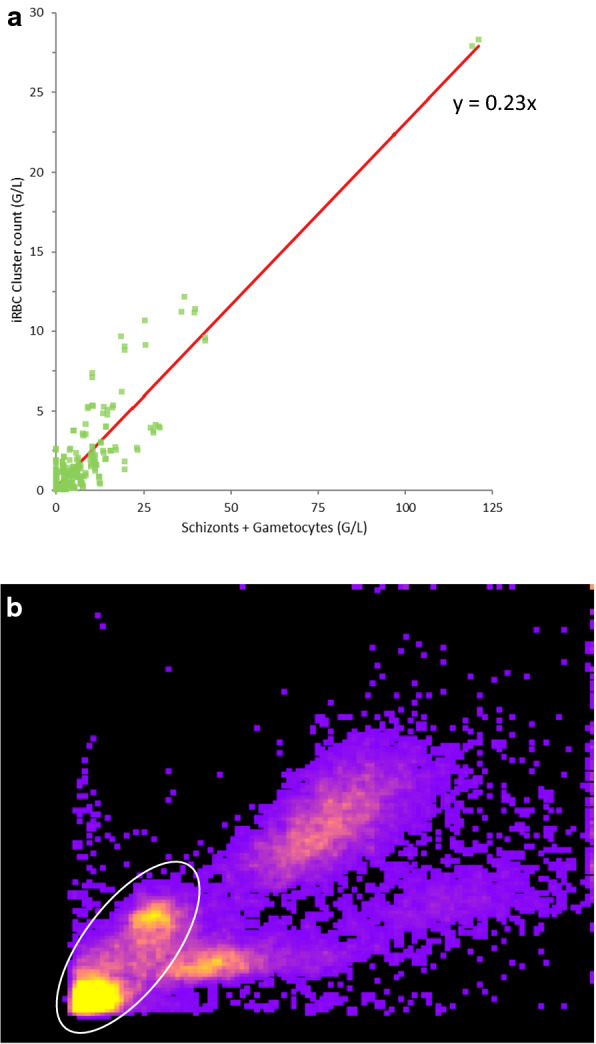
**a** Correlation of iRBC cluster counts *vs* microscopic schizonts + gametocytes counts. **b** Screen shot of density plot showing double iRBCs clusters seen in a subset of malarial cases

The malaria cases presenting with abnormal scatter events as an iRBC cluster of the WBC matrix (Fig. 1) were further investigated to correlate with the presence of specific malarial parasite forms. The size of these clusters was computed from the proportion of cellular events for 100 WBCs and WBC counts, and correlated with the microscopic counts of malarial parasite forms per 2000 RBCs extrapolated for the RBC counts from the Analyzer. No correlation was found for ring forms nor amoeboid forms of *Plasmodium* with the iRBC counts. However, a strong correlation (R^2^ = 0.893) was found between the most mature forms of the *Plasmodium* (schizonts and gametocytes) and these iRBC events in the WBC matrix (Fig. 6a).

This suggests that only these mature forms are large enough to be detected by the optical plus impedance technology of the Yumizen H550. It is also possible that these mature forms have remodeled the iRBC cytoskeleton resulting in increased lyse resistance. Surprisingly, the slope of linear model was about 0.2, suggesting that only 20% of these mature forms are seen in the WBC matrix and suggesting only a subset of parasitized RBCs are lyse resistant.

A few samples showed two distinct clusters of iRBCs (Fig. 6b). In some of these samples, the upper right cluster was higher in density than the lower left one, ruling out doublets to be the cause. Neither platelet clumps nor macro-platelets were seen on the corresponding slides. This was also observed in some samples where the predominant forms were rings and schizonts. Presently the exact interpretation of these two clusters remains unclear and a subject of further study.

Although the dengue fever flag performance was lower than that for the vivax malaria flag, it is worthwhile to note that currently there is no reported specific pattern of dengue infection by any standard commercial Haematology CBC-DIFF Analyzer. The machine learning process however was able to build a model performing a relatively efficient screening indicator for dengue infection. This suggests that the machine learning process has extracted many subtle but parallel variations in cell measures resulting from the unique immune response of patients with dengue fever. These findings suggest further investigative ‘profiling’ of various other viral diseases might be equally productive.

These findings are very encouraging for the feasibility of an affordable commercial malaria and dengue infection screening flag on cost-effective Haematology Analyzers, but still represent a single site study of an ethnically-biased population with a predominance of one malarial species. Profiling the immune response of the patient might also lead to diagnostic pitfalls or limitations, because the response may not be universal and vary from location to location depending on the ethnicities and genetics of patients and/or genetic variations of pathogens. Another limitation of this study is that the performance evaluation was carried out at a single location with a limited number of instruments, albeit that the same instrument was not used to build the models for the malarial and dengue fever flags as that used in the initial performance evaluation.

## Conclusions

For the first time with Haematology Analyzers using cost-efficient technology, the HORIBA Medical Yumizen H550 demonstrates flags for vivax malaria and dengue fever that can be clinically useful for the screening of these infections in low-resourced, endemic areas and thus facilitate further diagnosis testing in a cost effective manner.

Unlike previously reported malarial and dengue screening studies using only high range instruments [7–9, 16–20, 23–27], this approach is based on machine learning from instrument generated raw data measurements from a more affordable CBC-DIFF analysis and does not depend on prior cell population classifications of the sample. This ability to screen for diseases like dengue fever without a unique disease specific signal suggests that machine learning data-mining techniques can provide means of disease profiling based upon the immune or cellular responses of the patient. This approach with continued refinement could be extended to screen for various other pathologies and performance could be further improved as the experiential database increases.

## Data Availability

The datasets used and/or analyzed during the current study are available from the corresponding author on reasonable request.

## Abbreviations

RBC: Red blood cell
iRBC: Infected red blood cell
CBC: Complete blood count
DIFF: Leucocytes differential
WBC: White blood cell
ROC: Receiver operating characteristic
AUC: Area under curve
CI: Confidence interval
PLT: Platelets
PPV: Positive predictive value
NPV: Negative predictive value
RDT: Rapid diagnostic test
CRP: C-reactive protein
